# A Probabilistic Approach to Receptive Field Mapping in the Frontal Eye Fields

**DOI:** 10.3389/fnsys.2016.00025

**Published:** 2016-03-18

**Authors:** J. Patrick Mayo, Robert M. Morrison, Matthew A. Smith

**Affiliations:** ^1^Department of Neurobiology, Duke UniversityDurham, NC, USA; ^2^Center for the Neural Basis of Cognition, University of PittsburghPittsburgh, PA, USA; ^3^Center for Neuroscience, University of PittsburghPittsburgh, PA, USA; ^4^Medical Scientist Training Program, University of PittsburghPittsburgh, PA, USA; ^5^Department of Ophthalmology and Department of Bioengineering, University of PittsburghPittsburgh, PA, USA; ^6^Fox Center for Vision Restoration, University of PittsburghPittsburgh, PA, USA

**Keywords:** frontal eye field, prefrontal cortex, vision, generalized linear model, saccade, oculomotor, remapping, macaque

## Abstract

Studies of the neuronal mechanisms of perisaccadic vision often lack the resolution needed to determine important changes in receptive field (RF) structure. Such limited analytical power can lead to inaccurate descriptions of visuomotor processing. To address this issue, we developed a precise, probabilistic technique that uses a generalized linear model (GLM) for mapping the visual RFs of frontal eye field (FEF) neurons during stable fixation (Mayo et al., 2015). We previously found that full-field RF maps could be obtained using 1–8 dot stimuli presented at frame rates of 10–150 ms. FEF responses were generally robust to changes in the number of stimuli presented or the rate of presentation, which allowed us to visualize RFs over a range of spatial and temporal resolutions. Here, we compare the quality of RFs obtained over different stimulus and GLM parameters to facilitate future work on the detailed mapping of FEF RFs. We first evaluate the interactions between the number of stimuli presented per trial, the total number of trials, and the quality of RF mapping. Next, we vary the spatial resolution of our approach to illustrate the tradeoff between visualizing RF sub-structure and sampling at high resolutions. We then evaluate local smoothing as a possible correction for situations where under-sampling occurs. Finally, we provide a preliminary demonstration of the usefulness of a probabilistic approach for visualizing full-field perisaccadic RF shifts. Our results present a powerful, and perhaps necessary, framework for studying perisaccadic vision that is applicable to FEF and possibly other visuomotor regions of the brain.

## Introduction

The maintenance of a stable visual world despite frequent eye movements is a critical function of the visual system. The brain must coordinate the processing of incoming visual stimuli with updated motor plans and ongoing changes in internal state. The neuronal mechanisms supporting this rapid and complex coordination are unclear.

One way to investigate this process is to focus on changes in the visual sensitivity of single neurons around the time of an eye movement (Duhamel et al., 1992; Walker et al., 1995; Kusunoki and Goldberg, 2003; Sommer and Wurtz, 2006). But this approach is limited by the rate at which the visual field is sampled; the finest discriminable changes in receptive field (RF) structure correspond to the frequency with which RFs are probed. Spatial visual sensitivity has typically been coarsely measured in oculomotor regions of the brain (e.g., Goldberg and Wurtz, 1972; Sommer and Wurtz, 2006) in contrast to the detailed spatial characterizations of RFs in early visual cortex (Jones and Palmer, 1987; Reid et al., 1997; Ringach et al., 1997; Rust et al., 2005). As a result, our understanding of perisaccadic vision and the underlying circuit mechanisms has been limited.

To increase the resolution of RF measurements in the frontal eye field (FEF), we developed a probabilistic approach for quantifying and visualizing full-field RFs at timescales as fine as tens of milliseconds (Mayo et al., 2015). We previously visualized RF dynamics during stable fixation and found that: (1) conventional and probabilistic measures of FEF RFs produced similar results (cf., Churan et al., 2011); (2) we could track the visual responsiveness of FEF neurons at rates up to every 10 ms; and (3) we could produce detailed maps of the entire RF, including its size, approximate shape, and center. These results suggest that probabilistic mapping is a promising avenue for measuring perisaccadic RFs in FEF.

Although we previously demonstrated the usefulness of probabilistic RF mapping and the relationship of this method to conventional RF mapping, it is important to understand the limitations of the approach in the context of particular choices regarding the experimental design and visual stimulus parameters. These practical concerns are crucial for any newly developed technology, and we therefore detail the topics that are likely relevant to researchers below. To help determine the ideal stimulus parameters for measuring visual RFs in FEF, we measure changes in the quality of RF model fits as a function of number of trials and recording time. Although our prior work (Mayo et al., 2015) found few differences in RF quality between conditions and therefore suggested using dense and rapid stimuli, our more robust analyses here find that it is best to present a few stimuli (1 or 2 dot stimuli per video frame) stochastically at relatively slow presentation rates (150 ms per video frame). Because randomly presented stimuli unevenly sample visual space when trials counts are low, we also evaluated the effect of such biased sampling and present a straightforward way of dealing with under-sampled stimulus locations in the RF. We extend our approach to detail the fine spatial structure of responsive FEF neurons and probe the limits of the spatial resolution of probabilistic mapping. Finally, we illustrate the potential of our approach by characterizing RF changes in an example FEF neuron around the time of a saccade.

## Materials and Methods

Our methods and dataset are the same as those reported in detail in Mayo et al. (2015), except for Figure 6 (see below). Our prior work found similar results in two adult male rhesus macaque monkeys (*Macaca mulatta*). But, we only tested a broader range of stimulus parameters in one monkey and those are the neurons which we analyze in more detail here (*n* = 61 neurons). It is important for future work to validate these results in additional animals. All procedures were approved by the Institutional Animal Care and Use Committee of the University of Pittsburgh and complied with guidelines set forth in the National Institutes of Health (NIH) *Guide for the Care and Use of Laboratory Animals*.

We used single electrodes to isolate visually-responsive neurons in FEF, located in the anterior bank of the arcuate sulcus, and functionally identified FEF by using electrical microstimulation (Bruce et al., 1985). During probabilistic mapping, the monkey maintained central fixation on a small blue circle for 3 s during each trial while sparse, stochastic stimuli (white dots on a black background) were presented. The number of square dot stimuli (1, 2, or 8 dots per stimulus presentation) or the frame rate of stimuli presentation (10, 30, 70, or 150 ms per frame) were varied across trials. Dot locations were chosen randomly, and the dots were scaled to increase in size with eccentricity to approximate the magnification factor of V1 RFs (Dow et al., 1981). Monkeys were rewarded for maintaining fixation until the fixation point was turned off at the end of the trial. Aborted trials were excluded from the analyses.

Spike trains recorded from individual neurons during probabilistic RF mapping were related to the time and location of visual stimuli using a generalized linear model (GLM; Pillow et al., 2008) and the “glmnet” toolbox in MATLAB (Friedman et al., 2010). Specifically, we used fivefold cross-validated, L1-norm lasso regression. The predicted spiking of the model was related to the beta values of the GLM by the equation:

$$Y_{t}=e^{\beta_{0}\;+\;\beta_{i}\;*\;X(i,t)}$$

where *β_i_* is the vector of coefficients fit by the GLM, *β_0_* is a baseline offset, *X* is a matrix of *i* pixel values (0 or 1) by *t* image presentations, and *Y_t_* is the predicted spike count of the model for image presentation *t*. GLM performance was calculated as the correlation (Spearman’s rho; ρ) between the predicted (*Y_t_*) and observed spike counts for each image presentation across all trials, averaged across the five cross-validated models. When averaging across cross-validations or neurons, Spearman’s ρ values were converted to *Z*-scores with the Fisher *r* to *Z* transformation prior to averaging, and then converted back to ρ. Spike counts were calculated in a time window with a latency and duration set to be optimal for each neuron for each condition (mean latency: 47 ms; mean duration: 114 ms) as described in Mayo et al. (2015).

In principle, we could run the GLM at the full resolution of the video monitor (1024 × 768 pixels), yielding 786,432 *β* values, and rely on regularization to compensate for data limitations. In practice, such a model is not realistic given current computing limitations and is not necessary given the spatial resolution of FEF RFs. We dealt with this issue by down-sampling the screen resolution to create “super-pixels”, each of which contained an equal number of actual screen pixels. Each super-pixel was assigned a value of 1 on a given image presentation if it contained an illuminated (white) pixel in the full image, and 0 if it did not (Mayo et al., 2015). In our previous report, we down-sampled the screen to a 32 × 24 resolution yielding 768 super-pixels, each ~1.9° square. We also set the degrees of freedom of the GLM as 20% of the total number of superpixels, which restricted the maximum proportion of non-zero *β* coefficients to 20%. Adjusting this parameter in the glmnet package sparsifies the resulting *β* coefficients.

In the current report, we systematically vary the down-sampled resolution and the GLM degrees of freedom. These two parameters were fixed values in our previous investigation. First, we vary the amount of down-sampling from 12 to 49, 152 super-pixels (4 × 3, where each super-pixel is ~14° square, to 256 × 192, where each super-pixel is ~0.2° square) and evaluate model performance. The lowest end of this range results in spatially coarse RFs and the upper end is computationally taxing and exceeds the spatial resolution of FEF neurons. The degrees of freedom of the GLM had little impact on the computation time in the range we tested (0.01, or 1% of *β* coefficients were permitted to be non-zero up to 0.5, or 50%).

For one neuron recorded from a second adult male rhesus macaque, we presented the stochastic dot stimulus in the context of a visually-guided saccade task. The monkey was trained to maintain fixation for 500 ms on a central blue circle, after which the fixation point was extinguished and a peripheral target simultaneously appeared. The monkey was rewarded for making a saccade to the target and maintaining fixation at the new location for an additional 500 ms. A single dot stimulus was shown at a new, randomly selected location every 30 ms throughout the duration of the trial. Spike counts were calculated in a time window from 60 to 140 ms after the onset of each dot image. Three independent GLMs were fit to these data, each with non-overlapping spiking activity: (1) the pre-saccadic model was fit from the images and spikes prior to the appearance of the saccade target; (2) the perisaccadic model was fit from the images and spikes in a period starting at the target appearance and ending 50 ms after the monkey’s eyes reached the target; and (3) the post-saccadic model was fit from the images and spikes in a period from the end of the perisaccadic window until the end of fixation (i.e., the end of the trial). The additional 50 ms after the saccade offset in the perisaccadic period was less than the visual latency of this neuron, and thus permitted the model to use the maximum amount of data to measure perisaccadic RF structure. The results were not qualitatively altered by ending the perisaccadic window immediately when the animal reached the saccade target.

## Results

We previously showed that sparse stimuli can be used to comprehensively map the visual sensitivity of FEF neurons during fixation (Mayo et al., 2015). This approach offers a number of improvements over previous RF mapping efforts in FEF and other visuomotor areas that presented one or two stimuli per trial (e.g., Umeno and Goldberg, 1997; Sommer and Wurtz, 2006). But improvements in spatial and temporal resolution often trade off with practical considerations like recording time and stable neuronal isolation. Below, we quantify the power and limits of probabilistic RF mapping to elucidate the issues researchers must address when measuring RF dynamics. We find that, on average, FEF neurons struggle to maintain spatial and temporal fidelity under very rapid (<100 ms) and somewhat dense (eight dots per image) stimulus conditions. As a result, the visual field may be improperly sampled in certain scenarios, a potential problem in any stochastic stimulus paradigm. We show that sampling issues can be somewhat ameliorated by smoothing or down-sampling visual space.

### Stimulus Presentation Rate

Our first goal was to determine whether visual activity in response to the presentation of many brief stimuli or fewer, long-duration stimuli per trial yielded better RF models. Our previous work (Mayo et al., 2015) evaluated RFs across conditions in terms of the maximum beta value for each GLM map and found no significant differences between conditions. Here, we re-evaluated the RF models in terms of “GLM performance” (ρ), which was the correlation between the cross-validated GLM’s predicted spiking activity and the observed spike train for a given neuron or neuron-condition combination (see “Materials and Methods” Section). This metric provides a clear and objective evaluation of the RF model—the best RF model is one which does the best job of predicting the neuronal response. If the temporal sensitivity of FEF neurons was unlimited, then clearly it would be better to present stimuli as rapidly as possible to densely sample visual space and minimize the number of trials needed. However, the temporal sensitivity of FEF visual responses is poorly understood. We recently found that we could obtain RF maps across a range of stimulus presentation rates, as high as 10 ms per frame (see Figure 5 in Mayo et al., 2015). Thus, we previously established that FEF neurons could, at a minimum, respond to rapidly presented visual stimuli.

But it is still unclear if RF models built on responses to fast presentation rates are better than those obtained at slower rates. Comparisons of model-derived RFs across stimulus presentation rates are complicated by the fact that we used a fixed trial duration of 3 s regardless of the presentation rate. Therefore different presentation rates led to differences in the total number of stimuli presented per trial. Changes in GLM performance across conditions could therefore be attributed to the presentation rate itself (temporal factors), differences in the density of spatial sampling (spatial factors), or a combination of the two.

To determine which factor had the larger influence on GLM performance, we focused on a subset of neurons in which we varied the stimulus presentation rate on a trial-by-trial basis (*n* = 27 neurons). A single dot stimulus was presented at 150, 70, or 30 ms per frame for the duration of a trial, resulting in 20, 42, or 100 stimuli presented per trial. For each neuron, we first fit a GLM to increasingly larger subsets of randomly sampled trials (from zero to the maximum number of trials collected, in increments of 10 trials). In a separate analysis, we randomly sampled 20 stimulus presentations per trial in each condition, again over an increasing number of sub-selected trials. A comparable analysis using subsets of trials in the order they were collected was used to test for changes in neuronal isolation over time; the results did not meaningfully differ from those obtained by random sampling presented below.

**Figure 1:**
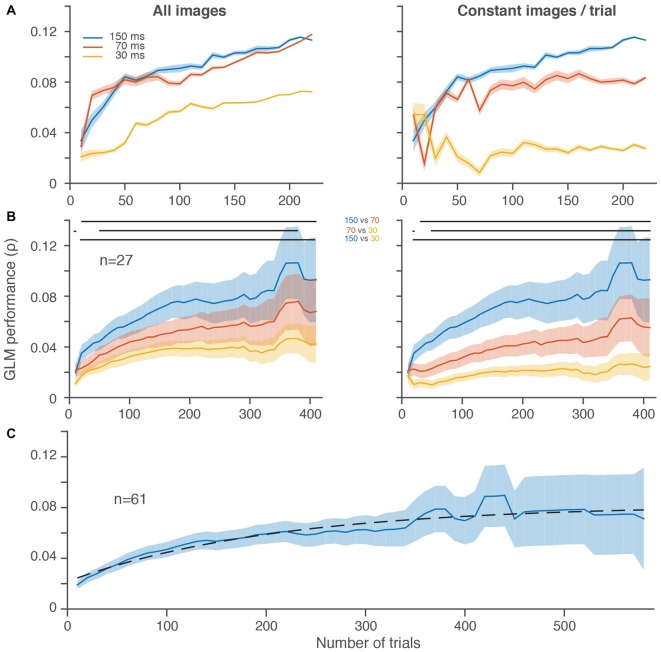
**Slower frame rates led to better generalized linear model (GLM) performance despite fewer stimulus presentations per trial. (A)** GLM performance over increasingly larger random subsets of trials for three different stimulus presentation rates in a single frontal eye field (FEF) neuron in 10 trial increments. Left, all stimulus image presentations are included without regard to the total number of stimuli per trial, which differed between conditions. Right, an equal number of stimulus image presentations (20, matched to the slowest stimulus presentation rate) was selected on each randomly sampled subset of 10 trials. **(B)** Average GLM performance per condition across neurons. The increase in noise (wider shading) from left to right is because fewer neurons contributed to the average at the higher trial counts. GLM performance for individual neurons decreased in noise with increasing trials (**A**). Right and left plots are same format as in **(A)**. In both **(A,B)**, the first subsample (10 trials) is omitted for display purposes. In **(B)**, the last five data points are omitted because there were less than 4 neurons with more than ~400 trials per condition. Horizontal lines at the top of the plots indicate significant differences (*p* < 0.05) between conditions based on paired *t*-tests at each resolution. **(C)** Average GLM performance across all 61 neurons, using the best condition for each neuron (see Mayo et al., 2015). Dashed black line is a fitted saturating exponential which summarizes the improvement in GLM performance as a function of the number of trials collected. Shading represents ± one SEM.

For the 150-ms stimulus presentation condition, the two analyses were identical by definition (e.g., the blue lines are the same in Figures 1A,B, left and right) because there were always 20 stimulus presentations per trial. For the 70 and 30-ms presentation rates, the second “constant images per trial” analysis controlled for the number of stimuli presented per trial and allowed us to isolate the temporal factors that contributed to GLM performance. If the quality of GLM fits was based only on the total number of stimuli presented (which correlates with sampling density), then we would expect the 30-ms condition to yield the best performance and the 150-ms condition to perform the worst.

Figure 1A shows the performance of a GLM built using three different stimulus presentation rates over the course of more than 200 trials for a single FEF neuron. Performance improved in all conditions as the number of trials included increased, as expected (Figure 1A, both panels). But, despite the fact that it probed visual space the fewest number of times, the 150-ms condition yielded the best performance after equating the number of image presentations per trial (Figure 1A, right). When the faster stimulus presentations were allowed to accumulate more images (Figure 1A, left), GLM performance greatly improved for the 70 and 30-ms conditions (compare red and yellow lines from Figure 1A, right vs. left). In the case of the 70-ms condition, the GLM performance rose to a level nearly identical to the 150-ms condition. Thus, for this neuron, it was possible to sample visual space as rapidly as every 70-ms, but a faster sampling of every 30-ms led to a substantially worse estimate of the RF.

In the population of FEF neurons recorded in this paradigm, we found a similar overall trend (Figure 1B). Average GLM performance improved as more trials contributed to the construction of the models. More importantly, activity from the 150-ms condition led to better performance even after including the increased number of stimuli presented with the faster 70 and 30-ms conditions (Figure 1B, left). Whereas the spiking activity of the single neuron example in Figure 1A (left) was similar for the 150 and 70-ms conditions, across the population of neurons there was a more gradual improvement in GLM performance from 30 to 70 to 150 ms (Figure 1B, right). Although we could successfully construct predictive GLMs at all presentation rates, the slowest rate that we used provided the best RF models.

To better estimate the number of trials needed to reach peak GLM performance, we performed a similar analysis on all stimuli presented in the best condition from all 61 neurons with a significant RF (Mayo et al., 2015) from the same monkey (Figure 1C). These data were best fit, in the least squares sense, with a saturating exponential function that had a trial constant of 208, demonstrating that on average 200 trials were sufficient to achieve more than 70% of the maximum GLM performance we observed. The fact that GLM performance tended to saturate in the trial ranges that we employed indicates that under-sampling is not an insurmountable issue in this regime.

### Spatial Resolution and Sampling

**Figure 2:**
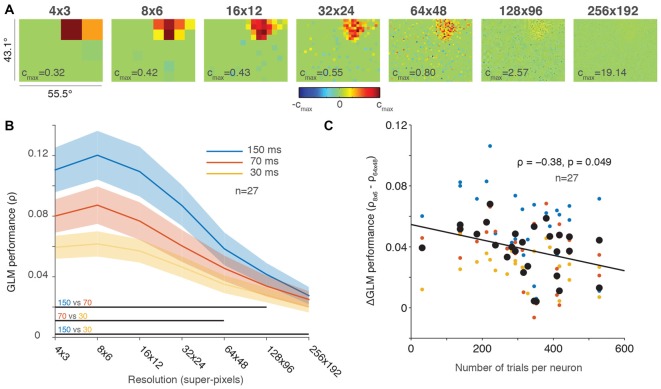
**Greater down-sampling generally yielded better GLM performance. (A)** An example neuron’s receptive field (RF) plotted using a range of screen resolutions. Note that the color bar for each plot is scaled to its maximum and all plots use a degrees of freedom equal to 20% of the total number of superpixels. **(B)** Population averaged GLM performance for each stimulus presentation rate (150, 70 or 30 ms) as a function of resolution. Shading represents ± one SEM. Horizontal lines at the bottom of the plot indicate significant differences (*p* < 0.05) between conditions based on paired *t*-tests at each resolution. **(C)** Change in GLM performance from low (8 × 6) to high (64 × 48) resolution (vertical axis) as a function of number of trials collected per neuron (horizontal axis). The black line is the least squares fit to the data. Small dots are color-coded to represent the performance for each condition (color convention in **B**) for an individual neuron; large black dots represent the average performance across conditions for each neuron.

Our prior work evaluated full-field RFs at a resolution of 32 × 24 “super-pixels” (Mayo et al., 2015) for computational expediency, which down-sampled the resolution of our 1024 × 768 pixel CRT video monitor. The middle panel labeled “32 × 24” in Figure 2A shows an example neuron from our previous work (same neuron as Figure 1D from Mayo et al., 2015). A strength of our probabilistic approach is that we can change the resolution of our RF maps, depending on our interests (e.g., RF sub-structure, computational expediency). Figure 2A shows the same neuron’s RF at seven different resolutions. The RF was clearly visible at resolutions up to and including 64 × 48 super-pixels and became faint at 128 × 96 super-pixels (note that the color scale of the RFs is normalized to the maximum *β* coefficient for each resolution, which increased with resolution). Thus, although the RF is difficult to visualize at higher resolutions and seemingly invisible at 256 × 192, some *β* coefficients remained, indicating large spiking responses due to image presentations at very focal locations. Our stochastic stimuli were presented at random locations on the screen, not in a pre-defined grid (e.g., Joiner et al., 2013). Therefore one drawback of this approach is that some pixels may be under-sampled, either presented too few times to contribute meaningfully to the GLM or never presented at all. This is of minimal consequence at lower resolutions where the neuronal responses to stimuli within a super-pixel are combined. But as the resolutions of the RF map increased, under-sampling was increasingly problematic (Figure 2A).

Figure 2B illustrates the same tradeoff between GLM performance and RF resolution in the population data. Consistent with our results in Figure 1, the 150-ms presentation rate yielded the best GLM performance across nearly all resolutions. The resolution with the best performance for all conditions was around 8 × 6 super-pixels, and performance gradually declined as the resolutions increased. While GLM performance was impaired at the highest resolutions by under-sampling, we are not aware of any previous work that measured full-field FEF RFs even at our lower resolutions such as 16 × 12 super-pixels, where each super-pixel was 3.5° square.

The population average in Figure 2B obscures the fact that we recorded a different number of trials for each neuron (usually, as many trials as stable neuronal isolation would allow; Mayo et al., 2015). If GLM performance decreased at higher resolutions solely because of under-sampling, then we would expect to see a smaller change in neurons where we recorded many trials and a larger change in neurons with fewer trials where under-sampling was greater. To investigate changes in GLM performance across resolutions as a function of trials collected, we calculated the difference in GLM performance between the 8 × 6 (low) and 64 × 48 (high) resolutions for each neuron. These resolutions were selected because they captured a range of resolutions where GLM performance varied but still resulted in reasonably precise fits (see Figures 2A,B). Figure 2C illustrates the change in GLM performance from low resolution to high resolution as a function of the number of trials collected. Although the size of our dataset was relatively small (*n* = 27), we identified a small negative correlation (*ρ* = −0.38, *p* = 0.049) between the change in performance and the number of trials per neuron. This result suggests that high-resolution RF maps can be acquired provided that sufficient numbers of trials are collected to offset under-sampling. Figure 2C therefore provides an estimate of the number of trials required to achieve the spatial resolution desired.

### Degrees of Freedom

GLMs are a powerful tool for measuring RFs in part because of regularization, which helps models cope with a large parameter space and limited data. In the lasso GLM employed here, the regularization serves to sparsify the parameter space. Explicit control over the sparsity of the *β* coefficients is achieved by setting the maximum degrees of freedom of the model (i.e., the maximum percentage of non-zero *β* coefficients). In the analyses above and in previous work (Mayo et al., 2015), we fixed the GLM degrees of freedom at 20% of the total number of super-pixels. Here, we quantify changes in GLM performance over a range of degrees of freedom while simultaneously considering the spatial resolution, to determine which values yielded the best performance.

**Figure 3:**
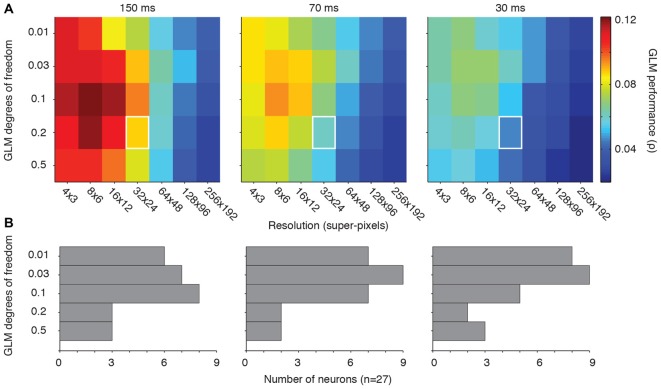
**Optimal GLM degrees of freedom. (A)** Matrices of GLM degrees of freedom as a function of down-sampling resolution in terms of average GLM performance for each of the three stimulus presentation rates. White squares represent the degrees of freedom (0.2) and resolution (32 × 24) used in our previous work (Mayo et al., 2015). **(B)** Distribution of the best degrees of freedom for each neuron in each condition.

Figure 3A shows the GLM performance as a function of the degrees of freedom (vertical axis) and resolution (horizontal axis). Our previous choice for GLM degrees of freedom, 0.2 or 20%, yields reasonable performance across all of the tested resolutions (Figure 3A, white squares). However, a value of 0.1 (or 10% of the super-pixels allowed to have non-zero *β* coefficients), yielded the best performance in all three conditions. Reassuringly, the range of penalties yielded consistent results within each resolution, and vertical slices of each condition’s matrix supported the conclusion from Figures 1, 2 that lower resolutions led to better performance. Thus, on average, the best choice for GLM degrees of freedom was 10% of the super-pixels across the population of neurons regardless of the stimulus presentation rate.

Figure 3B illustrates the optimal choice (in the terms of peak GLM performance) for degrees of freedom for each neuron across all resolutions. The “preferred” degrees of freedom varied widely and included all tested values. But the majority of neurons yielded their best GLM performance when the model degrees of freedom were relatively small. The diversity encountered here is consistent with the other metrics of FEF visual sensitivity, which tend to be heterogeneous across a given population (Sommer and Wurtz, 2000; Mayo et al., 2015). Some of this heterogeneity may stem from differences in the size of individual neurons’ RFs. The optimal degrees of freedom in the model should, in principle, be related to the proportion of the pixels occupied by the RF. This implies that both the resolution and degrees of freedom need to be adjusted to the characteristics of individual neurons for optimal model performance.

### Can Spatial Under-Sampling be Improved by Local Smoothing?

We have shown that there is a tradeoff between the density of sampling of visual space and the quality of a given RF map (Figures 1C, 2C). Sampling is a fundamental concern given our stochastic approach to stimulus presentation, especially when a large number of trials cannot be collected because of neuronal isolation or other issues. Thus, as fewer data are collected, the likelihood that a region of visual space is under-sampled increases. Figure 1 shows that it is possible to obtain sufficient RF measures in as few as 50 trials per condition (Figure 1A), and Figure 2 illustrates that lower resolutions yield the best results. However, one goal of the probabilistic approach is to obtain spatially precise RF measurements to evaluate models of perisaccadic circuitry. A spatially coarse RF, while sufficient in the sense of optimizing GLM performance, may be insufficient for such precision-dependent experimental questions. One potential way to “rescue” the performance of a high-resolution GLM is to use spatial smoothing.

**Figure 4:**
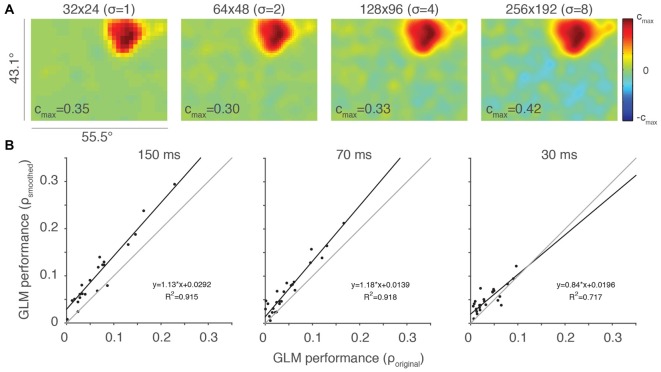
**Smoothing can help ameliorate under-sampling. (A)** Same example neuron from Figure 2A, here with Gaussian smoothing (SD = 2 super-pixels). The standard deviation of the smoothing kernel (*σ*) for each resolution is presented in parentheses. **(B)** Scatterplots of smoothed vs. original, unfiltered GLM performance for each of the stimulus presentation conditions (*n* = 27). Thin gray lines are lines of unity and black lines are least squares fits to the data.

We employed a Gaussian filter with a width proportional to the resolution of the RF, which kept the amount of filtering constant size in terms of visual space. The results of this filtering for the example neuron from Figure 2A at the four highest resolutions are shown in Figure 4 (32 × 24 resolution uses a Gaussian with SD = 1 super-pixels, 64 × 48 resolutions uses a Gaussian with SD = 2 super-pixels, etc.). By comparing the rightmost plots in Figure 2A to their smoothed counterparts in Figure 4A, it is clear that averaging beta values within a localized region of space helps recover details of the RF that were otherwise difficult to observe. This is particularly noticeable at the two highest resolutions, where a dim (128 × 96 super-pixels) RF and a nearly invisible (256 × 192 super-pixels) RF were made clear by the smoothing procedure.

We evaluated the impact of this Gaussian filtering at a resolution of 64 × 48 super-pixels, comparing the performance of the smoothed and original, unfiltered GLMs (Figure 4B). GLM performance was highly correlated between the smoothed and unsmoothed models, but consistently better in the smoothed case. This was especially apparent at the stimulus presentation rate of 150 ms, where under-sampling is more likely to occur. Thus, smoothing is helpful in cases where the RF is poorly sampled. Smoothing, of course, also reduces the salience of the features that the high-resolution sampling is intended to access. While it remains unclear if this type of smoothing will improve the ability of the experimenter to access these features, it is certainly a useful tool that provides flexibility in the experimental application of GLMs to RF mapping.

### Simultaneously Sampling Multiple Stimulus Locations

Except for Figure 1C, the results above focused on a sub-population of neurons (*n* = 27) in which we varied the stimulus presentation rate on each trial. These neurons were useful because they allowed us to determine that stimuli presented at a faster rate do not necessarily lead to better GLM performance. On average, RF maps were best for the slowest presentation rate tested (150 ms; Figure 1B). However, within each condition, more trials (i.e., more sampling) improved the quality of the GLM fits, as expected. These results demonstrate that the need to sample visual space densely is limited by the relatively sluggish temporal impulse response of FEF neurons.

Denser sampling of visual space can also be achieved by presenting multiple stimuli on each frame, instead of presenting a single stimulus at a faster rate. We previously recorded the responses of single FEF neurons to 1, 2, and 8 dot-stimuli per video frame (fixed within a trial) at a presentation rate of 70 ms per frame in a separate subpopulation of neurons (*n* = 20; Mayo et al., 2015). To determine if GLM performance improves when spatial sampling is increased on each video frame, we compared GLM performance across the population of neurons in each of the “differing number of stimuli per frame” conditions. Like the analyses in Figures 2B,C, 3A, these analyses did not use the smoothing methods illustrated in Figure 4.

**Figure 5:**
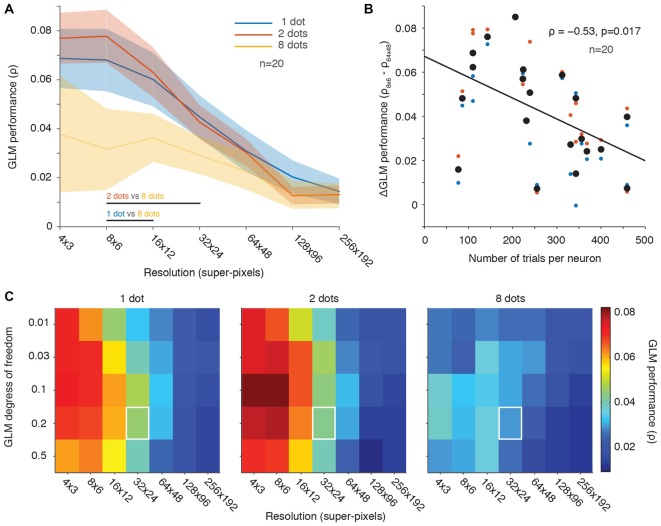
**Fewer stimuli per frame yielded better GLM performance than many stimuli. (A)** Population averaged GLM performance as a function of resolution (same format as Figure 2B). Each line represents the performance for a condition with a constant number of dot stimuli per video frame (1, 2 or 8). Shading represents ± one SEM. Horizontal lines at the bottom of the plot indicate significant differences (*p* < 0.05) between conditions based on paired *t*-tests at each resolution. No significant differences were observed between the 1 dot and 2 dots conditions at any resolution. **(B)** Change in GLM performance (same format as Figure 2C) from low to high resolution (vertical axis) as a function of number of trials collected per neuron (horizontal axis). The 8-dots data were excluded because of noisy fits across all resolutions (yellow line in **A**). The black line is the least squares fit to the data. **(C)** GLM degrees of freedom as a function of down-sampling resolution in terms of average GLM performance for each of the three stimulus conditions (same format as Figure 3A). White squares represent the degrees of freedom (20% of the number of superpixels) and resolution (32 × 24) used in our previous work (Mayo et al., 2015).

Figure 5A shows that the simultaneous presentation of multiple stimuli on each video frame did not improve GLM performance. Performance was actually poorest in the 8-dot condition where spatial sampling was greatest (Figure 5A, orange line). In contrast, one or two stimuli per video frame yielded comparable population averaged GLM performance that was better than that of the eight dots condition (Figure 5A, blue and red lines). Thus, GLM performance was not simply dependent on the spatial sampling. This result complements Figure 2B where we also found that denser temporal sampling (achieved by faster presentation rates) produced poorer model performance. We conclude that using faster stimulus presentation (Figure 2B) or more stimuli on each video frame (Figure 5A) does not lead to better GLM performance, contrary to predictions based solely on the density of RF sampling.

We next looked at the effect of the number of trials collected per condition as a function of GLM performance across low vs. high RF resolutions (same analysis as in Figure 2C). For this analysis, we excluded the data for the eight-dot condition because it was noisy and produced poor GLM performance (see Figure 5A). Figure 5B demonstrates that GLM performance was better at low resolutions than at high resolutions for neurons in which we collected fewer trials (Figure 5B). This was presumably because the high-resolution RF maps were under-sampled.

We also investigated whether changes in the GLM’s degrees of freedom or its spatial resolution improved GLM performance across the different-number-of-stimuli conditions (Figure 5C, analogous to Figure 3A). The relationship between the GLM’s degrees of freedom and its resolution was similar across conditions and similar to our previous observations for varying stimulus presentation rate (Figure 3A). But performance was better overall in the 1-dot and 2-dot conditions than in the 8-dot condition. Thus, as was the case in Figure 3A, the results were not dependent on the specific GLM penalty or resolution chosen, which were always kept constant across conditions. Overall, the 2-dot condition yielded the best GLM performance, followed closely by the 1-dot condition, while the 8-dot condition’s performance was noticeably diminished.

### Perisaccadic RF Mapping

Figures 1–3 demonstrated that relatively slow stimulus presentation rates (150 ms/frame) lead to better GLM performance. Figure 4 suggests that some amount of under-sampled data can be recovered by using local Gaussian smoothing, and Figure 5 shows that fewer stimuli per video frame (1–2 dots) yield better GLM performance that many dots per frame. Taken together, these results place strong constraints on the use of our probabilistic approach for perisaccadic RF mapping, which requires dense sampling in relatively brief epochs of visual activity. We tested the probabilistic approach’s usefulness for perisaccadic RF mapping by presenting stochastic sparse noise stimuli during a standard visually-guided saccade task. We illustrate the results in a single FEF neuron below (for details, see “Materials and Methods” Section).

**Figure 6:**
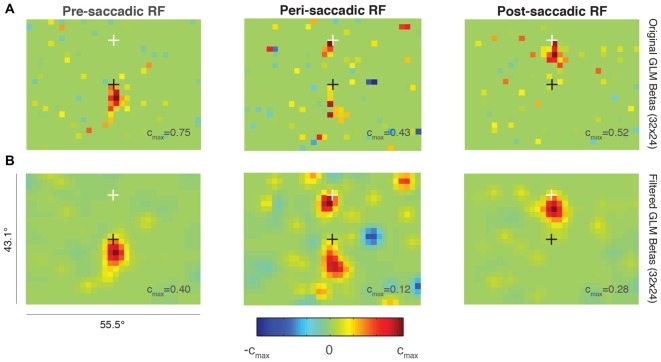
**Probabilistic perisaccadic RF mapping. (A)** A single FEF neuron’s RF before (left), around the time of (middle), and after (right) an upwards 15° saccade. The black cross indicates the initial fixation point and the white cross indicates the saccade endpoint. Note that each plot is scaled to its maximum beta value (*c_max_* value). **(B)** Same data and format as **(A)** but smoothed using a Gaussian of SD = 1 super-pixel.

Figure 6 shows the RF of an example FEF neuron around the time of a 15° upward saccade. The top row (Figure 6A) shows the unfiltered predictions of three independent GLMs at a resolution of 32 × 24 super-pixels. The left plot illustrates the size and location of the RF before the onset of the saccade (“pre-saccadic RF”). Compared to the RF from a different neuron plotted at the same resolution in Figure 2A (middle panel), the current RF was smaller, consistent with its position closer to the fovea.

The middle plot in Figure 6A shows the RF calculated from data taken after the saccade target was turned on to just after the eye reached the target (“peri-saccadic RF”). This epoch was chosen to maximize the number of perisaccadic spikes included in the GLM. The RF was divided between its pre-saccadic location yoked to the initial fixation point (black cross), and its post-saccadic location yoked to the saccade endpoint (white cross).

After the saccade target was acquired, the RF was measured again while the monkey fixated the white cross (Figure 6A, right; “post-saccadic RF”). During this epoch, the RF was no longer divided and it returned to its conventional retinal position, just below the fixation point. The size and location were comparable in the pre- and post-saccadic epochs, as expected. Data from the same epochs but smoothed using a Gaussian with SD = 1 are shown in the bottom row of Figure 6B. The smoothing highlights the perisaccadic RF dynamics found in the unfiltered GLM predictions in Figure 6A.

We found no evidence of increased visual sensitivity in the spatial region between the pre- and post-saccadic RF locations for this neuron (green values on the color map). Such a divided perisaccadic RF is consistent with previous work in FEF showing a discrete “jump” between pre- and post-saccadic stimulus locations (Sommer and Wurtz, 2006). This result did not depend on the GLM parameters selected (resolution, degrees of freedom) and it was also present when the pre-, peri-, and post-saccadic epochs were made equal in duration. More work is needed to validate this method, but this example suggests that probabilistic RF mapping is a viable option for measuring full-field RF perisaccadic dynamics in FEF, and possibly other visuomotor brain regions.

## Discussion

Because neuronal RFs are often considered the fundamental processing unit of visual perception, a common approach for investigating perisaccadic vision is to measure single RFs before, during, and after an eye movement. Conventional methods present one stimulus per trial and therefore probe one location in visual space every few seconds. Averaging activity over these long periods of time complicates studies of the neuronal mechanisms of perisaccadic perception. In this report, we further elucidated a new probabilistic approach for measuring FEF RF dynamics.

In Figure 1, we showed that the predictions of cross-validated GLMs built on FEF visual responses were best when the rate of stimulus presentation was slow (150 ms per frame; Figure 1B, left). This effect was even more striking when the total number of stimuli presented per trial was balanced between conditions (Figure 1B, right). Figures 2, 3 extend this result by showing that it remains true regardless of the amount of down-sampling used to build the RF (Figure 2B) and regardless of the amount of RF map “sparseness” built into the GLM construction (Figure 3A). Figure 4 illustrates that local smoothing of the GLM’s beta values helps improve RF maps, especially at high spatial resolutions. Figure 5 shows that increasing the number of dots presented per stimulus frame did not improve the quality of GLM RFs. Despite these physiological limitations on our ability to densely sample visual space with a high frame rate, we were able to obtain a remarkably precise spatial measurement of an FEF neuron’s RF in the ~200 ms period around the time of a saccade (Figure 6, middle column). This result is an encouraging preliminary step toward more precise and reliable estimation of RF structure around the time of eye movements.

Although our experiment was designed in part to optimize the stochastic stimuli, the limitations of GLM performance provide some insight into the limitations of FEF visual responses. Given a theoretically instantaneous impulse response, the ideal stimulus presentation rate for deriving a RF would be as fast as our hardware allowed. In practice, however, visual responses are less able to track fast stimulus changes as the distance from the sensory periphery increases. While retinal ganglion cells and LGN neurons respond briskly to a grating flickering at 10–20 Hz (Derrington and Lennie, 1982, 1984), the low-pass temporal frequency tuning of V1 is even sharper. V1 neurons exhibit greatly reduced responses to stimuli drifting at rates greater than 10 Hz (Hawken et al., 1996). This decrease in the low-pass cutoff continues in area V2 (Foster et al., 1985) and V3 (Gegenfurtner et al., 1997), but has been rarely measured outside of striate and early extrastriate cortex. Although it is difficult to directly compare our results to those using drifting gratings, our finding of peak model performance with stimulus presentation rates of 150 ms (6.7 Hz) likely reflects continued downward pressure in the low-pass cutoff as visual stimuli are processed beyond striate cortex. A recent investigation of the temporal characteristics of spiking activity across cortical regions supports the notion of a hierarchy of intrinsic timescale, with prefrontal cortex ranking as the slowest (Murray et al., 2014). However, the relatively rapid latencies of FEF neurons (Pouget et al., 2005; Mayo and Sommer, 2013), along with our finding of robust responses for some neurons to stimuli updating as fast as every 10 ms, is evidence that FEF may have a faster intrinsic timescale than the rest of prefrontal cortex. Anatomical connectivity of FEF to other cortical regions like V4 also defies the traditional hierarchical placement for FEF (Anderson et al., 2011).

Even within the temporal constraints of FEF responses, a dense spatial sampling of the RF with the stochastic noise stimulus would still be ideal for a linear RF. But we found that even a modest increase in the number of stimuli (8 dots per frame) greatly reduced the efficacy of the GLM in predicting spiking responses. This result strongly implicates nonlinearities in the spatial summation of FEF neurons. There are some hints of this effect in the literature. Schall et al. (2004) as well as Cavanaugh et al. (2012) reported suppressive effects beyond the classical RF. Although our experiments were not directly aimed at studying this phenomenon, the results suggest nonlinear spatial summation in FEF RFs.

In broader terms, the apparent limits of FEF visual processing are consistent with FEF’s presumed role in cognitive control. One hypothesis, supported by our previous work (Mayo et al., 2015), is that FEF neurons may not be suited for ultra-rapid and multi-focused stimulus tracking. This “limitation” may therefore, instead, be a useful feature for maintaining or updating a salient visual stimulus (Thompson and Bichot, 2005; Joiner et al., 2011). It is also consistent with the relatively long timescale required to change the locus of spatial attention (Posner, 1980; Carlson et al., 2006; Herrington and Assad, 2010), which is important given FEF’s postulated role in attentional control (Schall, 1995; Clark et al., 2015). Our task did not manipulate the saliency of any particular visual stimuli, but our paradigm can be adapted to test this hypothesis in future work.

Our initial goal was to sample visual space in an even and minimally biased fashion, covering the largest possible area. While the manipulation of our stochastic dot stimulus was limited in its ability to improve RF estimation, there are a number of extensions that could prove fruitful. One direction would be to reduce the area over which the stochastic stimuli are shown, and thus sample a smaller spatial region. Another possibility is to manipulate the temporal structure of the dots such that spatially adjacent regions are not sampled close together in time. Finally, our chosen stimulus was a white dot on a black background, which was high contrast but otherwise not distinctive. Other shapes or combinations of contrasts may be more effective in different experimental contexts (Churan et al., 2011).

Regardless of the specific stimulus parameters used, our probabilistic approach provides a new way to classify previously overlooked properties of visually-responsive FEF neurons. FEF neurons have long been characterized by properties such as their relative visual and saccadic responsiveness (Bruce and Goldberg, 1985), their delay activity (Funahashi et al., 1989; Lawrence et al., 2005), and their response latencies (Pouget et al., 2005; Mayo and Sommer, 2013). These basic measures are undoubtedly important for understanding FEF. But other properties of FEF neurons (e.g., their preferred stimulus presentation rate or optimal degrees of freedom) may also help reveal how populations of FEF functions coordinate their activity. For example, even though our neuronal sampling was too sparse for such an analysis, it is possible that a larger, systematic sampling of neurons would reveal a relationship between neuronal properties and anatomical location. Such clustering of neuronal features, seen in primary visual cortex and in other visual areas (Mayo and Verhoef, 2014; Nienborg and Cumming, 2014), place important constraints on read-out mechanisms and suggest organized cortical or subcortical connections (Stanton et al., 1995; Sommer and Wurtz, 2000; Pouget et al., 2009). A comprehensive understanding of the spatial and temporal properties of individual neurons, and their interrelationships, may be needed to resolve the role of FEF in visual and cognitive processing.

## Author Contributions

JPM and MAS: designed the research. JPM, RMM, and MAS: collected data. JPM, RMM, and MAS: analyzed the data and wrote the manuscript.

## Funding

JPM was supported by NIH fellowship F32EY022529. RMM was supported by NIH grant T32GM008208. MAS was supported by NIH grants R00EY018894, R01EY022928 and P30EY008098, a career development grant and an unrestricted award from Research to Prevent Blindness, and the Eye and Ear Foundation of Pittsburgh.

## Conflict of Interest Statement

The authors declare that the research was conducted in the absence of any commercial or financial relationships that could be construed as a potential conflict of interest.
